# Inter- and Intra-Observer Agreement When Using a Diagnostic Labeling Scheme for Annotating Findings on Chest X-rays—An Early Step in the Development of a Deep Learning-Based Decision Support System

**DOI:** 10.3390/diagnostics12123112

**Published:** 2022-12-09

**Authors:** Dana Li, Lea Marie Pehrson, Lea Tøttrup, Marco Fraccaro, Rasmus Bonnevie, Jakob Thrane, Peter Jagd Sørensen, Alexander Rykkje, Tobias Thostrup Andersen, Henrik Steglich-Arnholm, Dorte Marianne Rohde Stærk, Lotte Borgwardt, Kristoffer Lindskov Hansen, Sune Darkner, Jonathan Frederik Carlsen, Michael Bachmann Nielsen

**Affiliations:** 1 Department of Diagnostic Radiology, Copenhagen University Hospital, Rigshospitalet, 2100 Copenhagen, Denmark; 2 Department of Clinical Medicine, University of Copenhagen, 2100 Copenhagen, Denmark; 3 Department of Computer Science, University of Copenhagen, 2100 Copenhagen, Denmark; 4 Unumed Aps, 1055 Copenhagen, Denmark

**Keywords:** artificial intelligence, chest X-ray, inter-rater, intra-rater, image annotation, diagnostic scheme, ontology, radiologists

## Abstract

Consistent annotation of data is a prerequisite for the successful training and testing of artificial intelligence-based decision support systems in radiology. This can be obtained by standardizing terminology when annotating diagnostic images. The purpose of this study was to evaluate the annotation consistency among radiologists when using a novel diagnostic labeling scheme for chest X-rays. Six radiologists with experience ranging from one to sixteen years, annotated a set of 100 fully anonymized chest X-rays. The blinded radiologists annotated on two separate occasions. Statistical analyses were done using Randolph’s kappa and PABAK, and the proportions of specific agreements were calculated. Fair-to-excellent agreement was found for all labels among the annotators (Randolph’s Kappa, 0.40–0.99). The PABAK ranged from 0.12 to 1 for the two-reader inter-rater agreement and 0.26 to 1 for the intra-rater agreement. Descriptive and broad labels achieved the highest proportion of positive agreement in both the inter- and intra-reader analyses. Annotating findings with specific, interpretive labels were found to be difficult for less experienced radiologists. Annotating images with descriptive labels may increase agreement between radiologists with different experience levels compared to annotation with interpretive labels.

## 1. Introduction

Plain chest X-rays (CXRs) are the most commonly used diagnostic image modality [1] and the first choice for most diseases of the lung, including pneumonia [2]. Hence, there is a large amount of CXRs every day for radiologists to interpret. With the worldwide shortage of radiologists and the continuing demand for CXRs, artificial intelligence (AI) and deep learning-based decision support systems have emerged as possible solutions to assist radiologists in the backlog of diagnostic images [3]. The large number of CXRs provides diverse information with varying complexity that is beneficial to the development and improvement of AI algorithms [4].

When developing an algorithm for a deep learning-based decision support system in radiology, developers need labeled images for training, validation, and testing [5]. Consistent labeling is a prerequisite for developing an effective algorithm [6]. Previous studies have suggested that variation in interpretation and denomination of CXR findings may be attributed to several factors, including the reader’s medical experience, terminology bias, local disease prevalence, and geographic location of the reader’s medical background [7,8]. Varying and inconsistent use of terminology, for whatever reason, may decrease the quantity of a given finding and complicate data preparation, which may render the algorithm ineffective.

Consistent labeling can be achieved by creating ontological systems for the annotation of diagnostic images. The importance of creating adequate ontological systems during AI development has previously been highlighted [9]. Several different ontological schemes for annotating CXRs have been developed, ranging from complex schemes with numerous labels to simple schemes consisting of only a handful of labels; PadChest [10] created a complex hierarchical labeling system with >180 unique labels, while Qure.ai [11] and CheXpert [12] had between 10 and 14 labels, respectively, for different chest-specific radiographic findings. Investigations on the construction of ontological schemes contribute to further insights into the challenges of creating suitable annotation labels for AI development [10].

As a step in data preparation for a novel deep learning-based decision support system, a customized diagnostic labeling scheme was developed. Instead of using already existing ontological schemes, customized labels were created to form our diagnostic scheme. The labels were made to be recognizable for Danish radiologists since they would annotate our final training, validation, and test datasets, which would consist of CXR images and text reports of Danish origin.

Our purpose was to collect information on clinicians’ behavior when using the diagnostic scheme and receive clinical feedback on the scheme’s construction and labels. Thus, this study’s main aim was to field test our diagnostic labeling scheme and evaluate the consistency of label use when radiologists of different levels of task experience annotated findings on CXR images. Our results could, in the future, be used to investigate how different deep learning algorithms perform depending on how the labels they used for training were ordered and/or categorized.

## 2. Materials and Methods

Ethical approval was evaluated and formally waived by the National and Regional Ethics Committee and Knowledge Centre on Data Protection Compliance due to the full anonymity of CXRs.

### 2.1. Diagnostic Labeling Scheme

**Figure 1:**
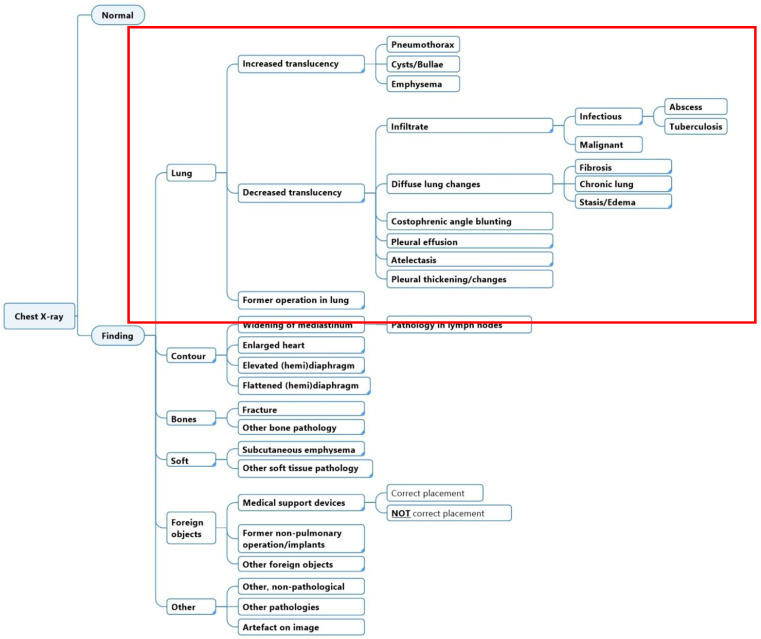
Full diagnostic labeling scheme and annotation labels for lung tissue findings in the diagnostic labeling scheme (red square).

The initial structure and labels in the diagnostic labeling scheme were generated with the aid of two radiologists. Labels were chosen based on a combination of the findings’ local clinical prevalence, urgency, and potential usefulness for clinicians. The goal of the scheme was that the sum of all labels should cover all the possible findings that are reported in a CXR. Furthermore, each label should be specific enough to be clearly differentiated from other labels and carry individual clinical meaning. Iterations and subsequent corrections were done in cooperation with a team of medical doctors, engineers, and data scientists. The diagnostic labeling scheme was evaluated to match existing collections of CXR ontology schemes or hierarchies, such as the Fleischner criteria and definitions [13], and other machine learning labeling strategies [10,11,12,14,15,16,17]. The annotation labels were represented in hierarchical classes, where a high-level class such as ‘Decreased translucency’ was divided into lower-level and increasingly more specific classes such as ‘Infiltrate’, ‘Pleural effusion’, etc. In this study, we investigated labels in the scheme related to lung tissue findings only (Figure 1).

### 2.2. Dataset and Annotation Software

**Figure 2:**
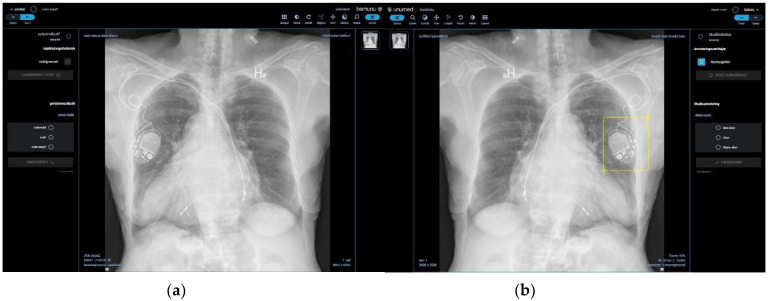
Image representations of the annotation software interface. (**a**) Front page layout of the annotation software and (**b**) bounding box for annotation of finding in the lower right hemithorax.

A selection of 100 fully anonymized CXRs were collected at the Department of Diagnostic Radiology at Rigshospitalet (RH) through the PACS system (AGFA Impax Client 6, Mortsel, Belgium) with the criteria that each label was to be represented in the corresponding text report in at least two cases. CXR images were imported to a proprietary annotation software program (Figure 2a,b) developed by Unumed Aps (Copenhagen, Denmark). Annotators were instructed to mark every single possible finding in both a lateral and frontal projection and select the most suitable annotation label.

### 2.3. Participants and Image Annotation Process

Six radiologists participated in the study. There were two radiologists at each experience level; novice radiologists with 1–2 years of experience; intermediate radiologists with 3–10 years of experience, and experienced radiologists with >10 years of experience.

**Figure 3:**
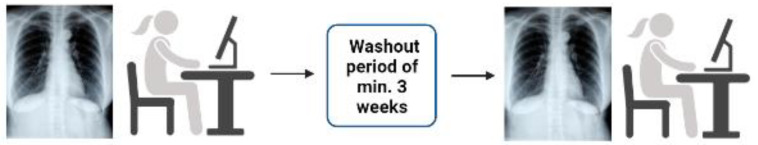
Visualization of the annotation process for each annotator.

Two blinded rounds of annotation were done, and no clinical patient characteristics were given. Rounds were interjected with a wash-out period of a minimum of three weeks from the last day radiologists had access to the CXR cases to the beginning of the second annotation round (Figure 3). Radiologists were allowed to contact the research and data scientist team for technical questions or difficulties. They were not allowed to share or discuss their annotations. No changes to the labels or the composition of the labeling scheme were made while the study ran its course.

### 2.4. Statistical Analysis

The inter- and intra-reader agreement using annotation labels from the diagnostic scheme on CXR data from Rigshospitalet has not been conducted prior to this study; thus, no formal sample or effect size computation was performed.

For each CXR case, a label would only appear to either have been used or not used for the statistical analysis, despite the label maybe having been used on both posterior–anterior and lateral projections of the same case. Continuous variables were reported in a frequency table.

Inter-reader agreement between all readers and between two readers of the same experience levels was done using data from the first annotation round. Randolph’s free-marginal multi-rater Kappa [18] was used to assess the overall degree of agreement between all participants. For two-reader inter-reader agreement between participants of the same level of radiological experience, prevalence-adjusted and bias-adjusted Kappa (PABAK) [19] was used. PABAK was also used to assess intra-reader agreement. Kappa is a commonly used chance-corrected statistic to measure the extent to which readers assign the same score to the same variable. Due to the possible unbalanced distribution of positive and negative labeled cases, we chose to use free-marginal Kappa as opposed to fixed marginal Kappa measurements. Kappa statistics were interpreted for strength by using the Landis and Koch scale [20].

Additionally, specific agreement, i.e., the proportion of positive agreement (PPA) and proportion of negative agreement (PNA), were calculated [19,21,22]. The PPA describes the shared number of cases in which a label was used out of the total number of cases where the label was used. The PNA describes the shared number of cases in which the label was *not* used out of the total number of cases that did *not* have that label. Analyses were done using RStudio Team (2021). RStudio: Integrated Development Environment for R. RStudio, PBC, Boston, MA URL http://www.rstudio.com (accessed on 2 July 2022), IBM SPSS Statistics for Windows, version 28.0 (IBM Corp. Released 2021, Armonk, NY, USA). Microsoft Excel 365 (2016) and an online kappa calculator were also used [23].

## 3. Results

Table 1 describes the number of CXR cases in which each label has been used in the first round of annotation. Novices used the broader and less specific label ‘Decreased translucency’ in 31–51 cases, while experienced radiologists did not use the label at all. However, experienced radiologists used the more specific label ‘Infectious infiltrate’ in 13–30 cases, while novice radiologists used it in only 0–2 cases. Intermediate radiologists also used the broader label ‘Infiltrate’ more often (24–33 cases) compared to the more specific label ‘Infectious infiltrate’ (3–6 cases). The novice and intermediate radiologists used ‘Diffuse pulmonary changes’ in 6–26 cases, while experienced radiologists only used it in 1 case. The majority of the radiologists marked between 11 and 19 cases as normal, except for one novice and one experienced radiologist, who marked 4 and 23 cases as normal, respectively.

### 3.1. Inter-Reader Agreement

#### 3.1.1. Agreement between Multiple Readers

All readers achieved *fair-to-excellent agreement* on all labels (Randolph’s Kappa, 0.40–0.99) (Table 2). ‘Atelectasis’ had the lowest agreement (Randolph’s Kappa, 0.40). Table 1 shows that an experienced radiologist marked 50 cases with ‘Atelectasis’, whereas the other radiologists marked between 9 and 25 cases. We did not differentiate between linear and segmental atelectasis either in the statistical analysis or in the annotation guidelines, which could explain the difference in frequency of use.

Congregate categories such as ‘Decreased translucency including sub-categories’ and ‘Costophrenic angle blunting AND pleural effusion’ reached the highest proportion of positive agreement (PPA) of 0.84 and 0.67, respectively. The congregate category ‘Infiltrate incl. sub-categories’ reached a PPA of 0.50, which is higher than any of its sub-categories. Otherwise, the only individual labels that reached a PPA above 0.50 were ‘Pneumothorax’, ‘Pleural effusion’, and ‘Normal’. However, all non-congregate labels reached a minimum of 0.81 in the proportion of negative agreement (PNA) (Table 2).

#### 3.1.2. Agreement between Two Readers with the Same Experience Level

There was *slight-to-excellent* agreement on all labels between radiologists of similar experience levels (Table A1 in Appendix A). The PABAK values ranged from 0.12 to 1.

The wide range in the PABAK values was most noticeable in the label ‘Decreased translucency’ where novices had the poorest agreement (PABAK 0.12), while experienced radiologists had the best agreement (PABAK 1). Table A2 (Appendix A) shows that the differences in agreement measures were due to the novice radiologists’ tendency to use this label more. Despite higher specific agreement on the positive use, it reduced the agreement on its negative use (PPA 0.46, PNA 0.63), while intermediate and experienced radiologists had no use of that label at all, resulting in very high specific agreement on the negative use (PPA 0 and PNA 0.94–1), which lead to the higher overall agreement.

Novice and intermediate radiologists also had a higher agreement on the positive use of the label ‘Infiltrate’ (PPA 0.48–0.53) (Table A2 in Appendix A), while experienced radiologists did not (PPA 0, PNA 0.84). Experienced radiologists had, however, higher agreement of the positive use of the more specific label ‘Infectious infiltrate’ compared to novice radiologists (PPA 0.14 vs. 0), despite having a lower overall agreement (PABAK 0.24 vs. 0.96).

Experienced radiologists showed *excellent* agreement on ‘Costophrenic angle blunting’ (PABAK 0.94), but only due to a high PNA and low PPA (PPA 0, PNA 0.98). However, all levels of radiologists agreed on the positive use of the label ‘Pleural effusion’ (PPA 0.47–0.86), and all levels of radiologists had a higher positive agreement on this label compared to ‘Costophrenic angle blunting’ (Table A2 in Appendix A). The congregate category ‘Costophrenic angle blunting AND pleural effusion’ also achieved a higher PPA compared to ‘Costophrenic angle blunting’ alone (PPA 0.64–0.72 vs. PPA 0–0.46).

Intermediate radiologists had a positive PPA on a greater number of labels compared to that of both novice and experienced radiologists (Table A2 in Appendix A), suggesting that intermediate radiologists used more labels overall. Despite this, all levels of radiologists had an equally good agreement on ‘Normal’ (PABAK 0.76–0.80), and intermediate radiologists generally had a comparable number of ‘Normal’ cases to the other radiologists (Table 1).

**Figure 4:**
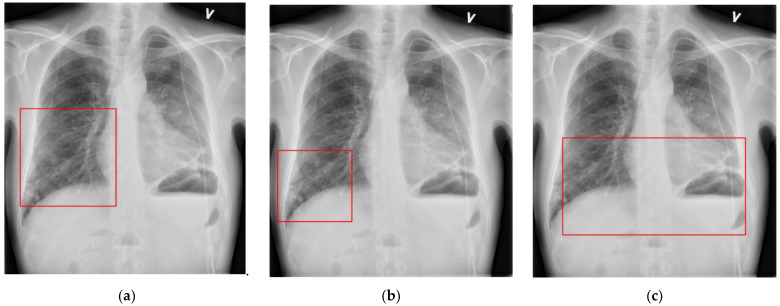
Examples of annotation bounding boxes labeled as (**a**) ‘Decreased translucency’ by a novice radiologist, (**b**) ‘Infiltrate’ by an intermediate radiologist, and (**c**) ‘Infection’ by an experienced radiologist on the same CXR case. Other findings and bounding boxes have also been used in this case but are not represented in this figure.

While novice radiologists had a higher specific positive agreement on broader and more unspecific labels, intermediate and experienced radiologists had a better specific positive agreement on more detailed and interpretive labels. Figure 4 shows an example of a similar finding on the same CXR case, labeled differently by a novice, intermediate, and experienced radiologist.

### 3.2. Intra-Reader Agreement

**Figure 5:**
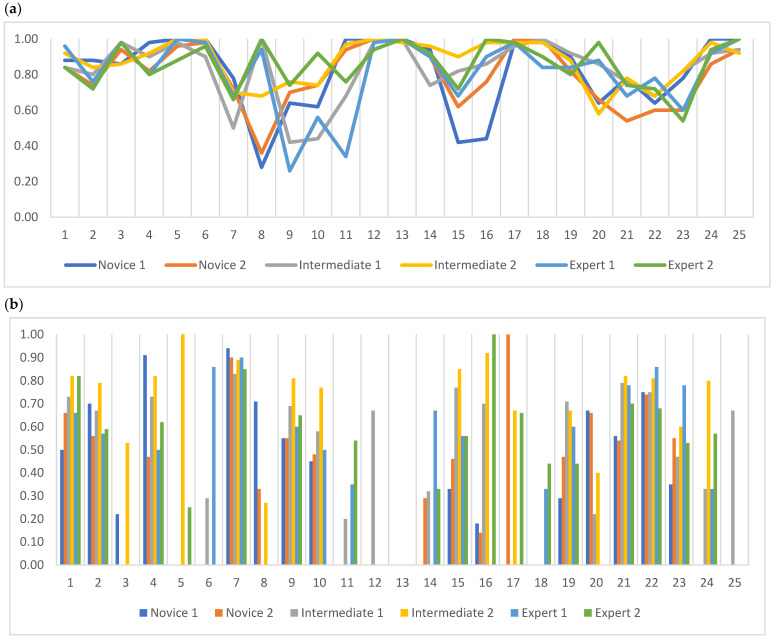
Intra-reader agreement measurements with (**a**) prevalence-adjusted and bias-adjusted Kappa (PABAK) and (**b**) proportion of positive agreement (PPA). 1. Normal, 2. Increased translucency incl. sub-categories, 3. Increased translucency, 4. Pneumothorax, 5. Cysts/bullae, 6. Emphysema, 7. Decreased translucency incl. sub-categories, 8. Decreased translucency, 9. Infiltrate incl. sub-categories, 10. Infiltrate, 11. Infection, 12. Abscess, 13. Tuberculosis, 14. Malignant, 15. Diffuse lung changes incl. sub-categories, 16. Diffuse lung changes, 17. Fibrosis, 18. Chronic pulmonary changes, 19. Stasis/Edema, 20. Costophrenic angle blunting, 21. Pleural effusion, 22. Costophrenic angle blunting AND pleural effusion, 23. Atelectasis, 24. Pleural thickening/changes, 25. Former operation in lung tissue. Kappa: <0, poor; 0.01–0.20, slight; 0.21–0.40, fair; 0.41–0.60, moderate; 0.61–0.80, substantial; 0.81–1.00, almost perfect.

All readers reached between 0.26 and 1 in the PABAK (Figure 5a), where ‘Decreased translucency’, ‘Infiltrate incl. sub-categories’, and ‘Infection’ had the lowest intra-reader agreement with PABAK values of 0.28, 0.26, and 0.34, respectively.

On specific agreement, all readers achieved over 0.50 in the PPA on ‘Normal’, ‘Increased translucency incl. sub-categories’, ‘Pneumothorax’, ‘Decreased translucency incl. sub-categories’, ‘Infiltrate incl. sub-categories’, ‘Pleural effusion’, and ‘Costophrenic angle blunting AND pleural effusion’ (Figure 5b). All readers reached between 0.52 and 1 in the PNA on all labels, with the lowest PNA on the label ‘Decreased translucency incl. sub-categories’ by one novice reader.

## 4. Discussion

The main findings of our study were that (1) simple, descriptive, and definitive labels reached greater specific positive agreement among readers with different radiological experience levels, (2) radiologists with less experience more often used and agreed on broader, unspecific labels compared to more experienced radiologists, and (3) the congregation of labels into broader categories increased the agreement for the same radiologists on two separate occasions.

Rudolph et al. [24] found the highest inter-reader agreement on pneumothorax and the lowest agreement on suspicious nodules. This resonated with Christiansen et al. [25], who showed the best performance in detecting pneumothorax and the worst in pneumonic infiltrate amongst a group of junior doctors. In concordance with these studies, our study showed that descriptive and definitive radiological diagnoses, e.g., pneumothorax or pleural effusion, which required nearly no additional patient information, were easier to detect and annotate, resulting in a higher specific positive agreement for all levels of radiologists, compared to interpretive diagnoses, such as infectious infiltrate [26]. Several deep learning solutions have been proposed to assist in the detection of infectious infiltrates [27,28], but due to the lack of consistent image annotation, our study suggests that such solutions must base their training data on multiple sources of information [10]. The integration of multiple sources of information to train an algorithm would be more time-consuming and costly, which could be the reason why several commercially available products have marketed AI-based systems for simple or descriptive findings on CXRs [29,30,31]. However, further studies are needed to examine the use of such solutions compared to solutions that aid in more interpretive radiological findings.

The strength of our study was the hierarchal layout of our diagnostic scheme. A previous study showed that label extraction following a hierarchical taxonomy increased labeling accuracy and reduced missing annotations [32]. Therefore, even with annotators with different radiological experience levels, there was less risk of missing data due to the option of labeling with a parent label instead of not labeling the finding at all. The hierarchical layout enabled us to analyze the differences in annotation between annotators with different radiological experience levels. Our study showed that experienced radiologists had greater confidence in labeling specific findings, e.g., ‘Infectious infiltrate’ vs. its parent label ‘Infiltrate’. However, novice radiologists were aware of the presence of an infiltrate but did not find confidence in specifying that finding and, therefore, used broader labels such as ‘Decreased translucency’ or ‘Infiltrate’. We showed that novice radiologists had enough training to enable them to recognize a pathological CXR from a normal CXR, but additional clinical training contributes to more confidence and refined recognition skills and detail orientation [33,34].

In terms of AI development, the different annotation behavior due to radiological experience can be used when recruiting data annotators. Our study suggested that the selection of annotators may be dependent on the annotation methodology. If annotations are on simple or broadly defined findings, less experience may be sufficient. However, if annotations on CXR images of complex diagnoses need to be made, our study suggested that more experienced radiologists were needed. It would be optimal to always have an experienced board-certified radiologist as an annotator [34]. Due to difficulties in the recruitment of highly specialized radiologists, AI development projects turn to annotators that are not radiologists [35]. Therefore, every AI development project needs to match the annotation methodology to the annotator’s experience to minimize time and cost while preserving accurate and consistent annotation.

Previous studies have shown that readers with less radiological task experience had poorer interpretation skills of diagnostic images compared to more experienced readers [36,37]. In our study, the positive agreement of fewer labels among novice radiologists could, therefore, be due to a lack of radiological experience. Although intermediate radiologists had a positive agreement on a greater number of different labels than the experienced radiologists, it did not result in fewer ‘Normal’ cases, which suggested that intermediate radiologists tended to over-annotate a single CXR case. This could have been due to either lack of task experience or a fear of missing diagnoses.

A bias in the study was the annotation process itself. The annotation process differs significantly compared to the radiologists’ normal free-text reporting, and the choice of annotation labels might be affected. All radiologists were given no clinical information on the cases, which could have been another bias in image interpretation. However, previous studies have not been conclusive in the benefits of additional clinical information on radiologists’ interpretive performance of CXRs [38,39].

The study was limited by the number of annotators and included cases. The limited number of cases affected the prevalence and distribution of the labels in the dataset because of natural prevalence patterns in the general population from which the CXR cases were obtained. Kappa statistics are dependent on prevalence. Since Kappa statistics is the agreement compared to chance, studies will inherently return a lower Kappa value if a label is either highly prevalent or highly un-prevalent in a dataset. We have provided the results adjusted for prevalence and bias (Randolph’s and PABAK) as a solution to the prevalence problem and as previously recommended [18,19,40]. In addition, deep learning algorithms cannot detect findings that are not there and, therefore, need to train on positively labeled data, which is why we also provided specific agreement measures, such as the proportion of positive agreement. Even though it is still possible for a high PPA when the prevalence is low, the likelihood of achieving a high PPA is low, which is why we reported both specific agreements and chance-adjusted agreements. Another limitation was that we did not test the performance of a deep learning solution that used the proposed labeling scheme as opposed to other labeling tactics. In this study, we, therefore, did not conclude whether our labeling scheme would create better-performing deep learning solutions when compared to deep learning solutions using other labeling schemes. We focused mainly on investigating agreement among radiologists as annotators when using our labeling scheme to annotate CXR image findings.

**Figure 6:**
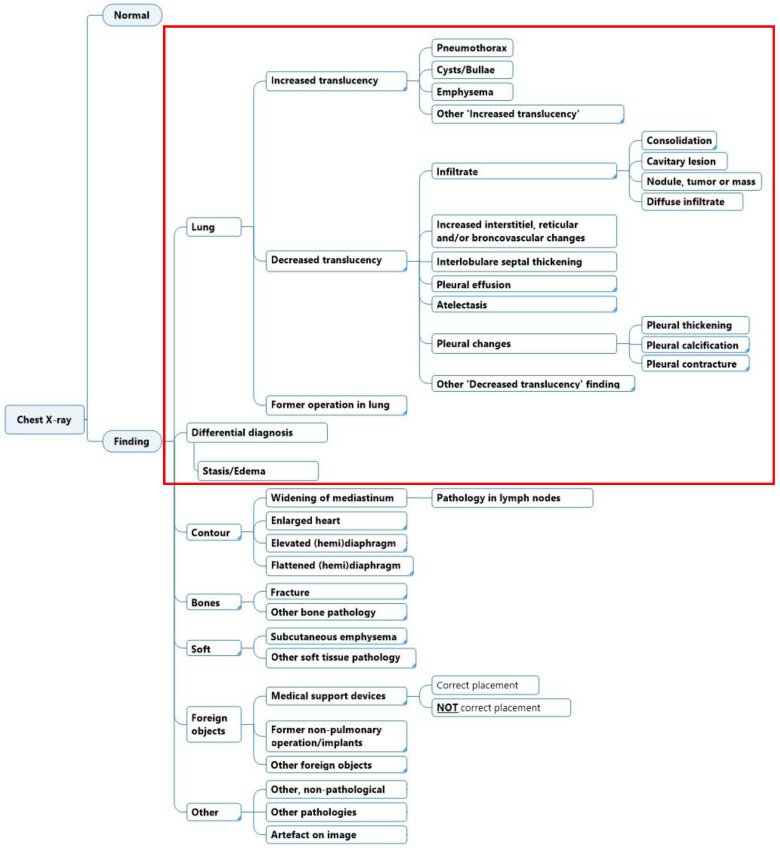
Proposed diagnostic labeling scheme for lung tissue findings (red square) on chest X-ray where interpretive labels have been replaced with more descriptive labels (corresponds to the labels encased with a red square in Figure 1).

This is the first study to investigate the inter- and intra-reader agreement when annotating CXR images for the purpose of developing a deep learning-based diagnostic solution. The annotators used bounding boxes when annotating findings to train the deep learning algorithms, but in our study, we did not specifically investigate whether the labeled finding was marked in the same location on the image since it was beyond the scope of this paper. For future perspectives, we suggest revising the diagnostic labeling scheme to include more descriptive labels to potentially increase positive agreement on lower-level labels for radiologists of different levels of task experience (Figure 6). Further studies are needed to investigate inter- and intra-reader agreement when using the suggested revised diagnostic scheme, as proposed in Figure 6.

## 5. Conclusions

Readers achieved *fair-to-excellent* agreement on all labels in our diagnostic labeling scheme. Differences in specific agreement showed a tendency to be dependent on radiological experience when distinguishing between using simple, descriptive labels or more complex, interpretive labels. However, further studies are warranted for larger datasets with a higher prevalence of both descriptive and interpretive findings.

## Figures and Tables

**Table 1:** Frequency table for each individual participating radiologist. The total number of cases out of 100 CXRs that had been annotated with that specific label by a radiologist. * Does not differentiate between linear and segmental atelectasis, which could explain the difference in frequency of use.

Lung Tissue Findings	Novice 1	Novice 2	Intermediate 1	Intermediate 2	Experienced 1	Experienced 2
Normal	4	11	11	19	11	23
Increased Translucency	7	3	8	0	0	0
Pneumothorax	5	8	11	10	10	9
Cyst/Bullae	0	1	1	0	5	2
Emphysema	0	1	0	3	4	0
Decreased Translucency	51	31	11	0	0	0
Infiltrate	21	12	24	33	24	2
Infection	0	2	3	6	30	13
Abscess	0	0	0	1	0	3
Tuberculosis	0	0	1	0	0	0
Malignant	3	6	1	10	5	3
Diffuse Lung Changes	26	6	7	11	0	1
Fibrosis	1	2	2	2	1	2
Chronic Lung Changes	1	0	1	0	5	2
Stasis/Edema	5	7	9	6	10	9
Costophrenic Angle Blunting	31	21	24	5	3	0
Pleural Effusion	8	22	32	24	38	27
Atelectasis *	14	22	13	9	50	25
Pleural Thickening/Changes	0	7	3	5	3	4
Former Operation in Lung Tissue	0	5	3	5	0	0

**Table 2:** Agreement between all readers measured in Randolph’s Kappa, proportion of positive agreement, and proportion of negative agreement. Kappa: <0, poor; 0.01–0.20, slight; 0.21–0.40, fair; 0.41–0.60, moderate; 0.61–0.80, substantial; 0.81–1.00, almost perfect.

All (n = 6)	Randolph’s Free-Marginal Multirater Kappa	95% CI for Randolph’s Free-Marginal Multirater Kappa	Proportion of Positive Agreement	Proportion of Negative Agreement
Normal	0.79	0.71–0.86	0.59	0.94
Increased Translucency incl. sub-categories	0.73	0.64–0.81	0.47	0.92
Increased Translucency	0.88	0.83–0.93	0	0.97
Pneumothorax	0.83	0.76–0.91	0.53	0.95
Cyst/Bullae	0.98	0.95–1.00	0.1	0.99
Emphysema	0.95	0.91–0.99	0.05	0.99
Decreased Translucency incl. sub-categories	0.55	0.45–0.64	0.84	0.59
Decreased Translucency	0.46	0.38–0.55	0.13	0.84
Infiltrate incl. sub-categories	0.40	0.31–0.48	0.50	0.78
Infiltrate	0.49	0.40–0.58	0.34	0.84
Infection	0.67	0.60–0.75	0.11	0.91
Abscess	0.97	0.95–1.00	0	0.99
Tuberculosis	0.99	0.98–1.00	0	1
Malignant	0.87	0.82–0.93	0.33	0.97
Diffuse Lung Changes incl. sub-categories	0.54	0.45–0.63	0.40	0.85
Diffuse Lung Changes	0.70	0.62–0.78	0.12	0.92
Fibrosis	0.95	0.91–0.99	0.28	0.99
Chronic Lung Changes	0.94	0.90–0.98	0	0.98
Stasis/Edema	0.79	0.71–0.86	0.31	0.94
Costophrenic Angle Blunting	0.58	0.49–0.67	0.25	0.88
Pleural Effusion	0.61	0.51–0.71	0.61	0.87
Costophrenic Angle Blunting AND Pleural Effusion	0.53	0.43–0.62	0.67	0.81
Atelectasis	0.40	0.30–0.50	0.32	0.81
Pleural Thickening/Changes	0.88	0.83–0.94	0.20	0.97
Former Operation in Lung Tissue	0.94	0.90–0.98	0.04	0.98

## Data Availability

Not applicable.

## Appendix A

**Table A1:** Prevalence- and bfias-adjusted Kappa (PABAK) for novice, intermediate, and experienced radiologists. Kappa: <0, poor; 0.01–0.20, slight; 0.21–0.40, fair; 0.41–0.60, moderate; 0.61–0.80, substantial; 0.81–1.00, almost perfect.

PABAK	Novice 1 vs. Novice 2	Intermediate 1 vs. Intermediate 2	Experienced 1 vs. Experienced 2
Normal	0.78	0.80	0.76
Increased Translucency incl. sub-categories	0.66	0.80	0.78
Increased Translucency	0.80	0.84	1
Pneumothorax	0.82	0.90	0.82
Cyst/Bullae	0.98	0.98	0.96
Emphysema	0.98	0.94	0.92
Decreased Translucency incl. sub-categories	0.48	0.64	0.50
Decreased Translucency	0.12	0.78	1
Infiltrate incl. sub-categories	0.62	0.56	0.22
Infiltrate	0.66	0.46	0.48
Infection	0.96	0.86	0.24
Abscess	1	0.98	0.94
Tuberculosis	1	0.98	1
Malignant	0.90	0.82	0.88
Diffuse Lung Changes incl. sub-categories	0.28	0.70	0.54
Diffuse Lung Changes	0.36	0.84	0.98
Fibrosis	0.94	0.96	0.94
Chronic Lung Changes	0.98	0.98	0.86
Stasis/Edema	0.84	0.86	0.70
Costophrenic Angle Blunting	0.44	0.54	0.94
Pleural Effusion	0.68	0.84	0.66
Costophrenic Angle Blunting AND Pleural Effusion	0.50	0.56	0.62
Atelectasis	0.44	0.72	0.38
Pleural Changes	0.86	0.88	0.94
Former Operation in Lung Tissue	0.96	0.84	1

**Table A2:** Specific agreement for novice, intermediate, and experienced radiologists. PPA, Proportion of positive agreement; PNA, Proportion of negative agreement. Kappa: <0, poor; 0.01–0.20, slight; 0.21–0.40, fair; 0.41–0.60, moderate; 0.61–0.80, substantial; 0.81–1.00, almost perfect.

Specific Agreement	Novice 1 vs. Novice 2		Intermediate 1 vs. Intermediate 2		Experienced 1 vs. Experienced 2	
	PPA	PNA	PPA	PNA	PPA	PNA
Normal	0.27	0.94	0.67	0.94	0.65	0.92
Increased Translucency incl. sub-categories	0.19	0.91	0.69	0.94	0.56	0.94
Increased Translucency	0	0.95	0	0.96	—	1
Pneumothorax	0.31	0.96	0.76	0.97	0.52	0.95
Cyst/Bullae	0	0.99	0	0.99	0	0.99
Emphysema	0	0.99	0	0.98	0	0.98
Decreased Translucency incl. sub-categories	0.84	0.32	0.87	0.72	0.81	0.62
Decreased Translucency	0.46	0.63	0	0.94	—	1
Infiltrate incl. sub-categories	0.56	0.88	0.68	0.83	0.43	0.70
Infiltrate	0.48	0.89	0.53	0.81	0	0.85
Infection	0	0.99	0.22	0.96	0.14	0.76
Abscess	—	1	0	0.99	0	0.98
Tuberculosis	—	1	0	0.99	—	1
Malignant	0.44	0.97	0.19	0.95	0.25	0.97
Diffuse Lung Changes incl. sub-categories	0.25	0.76	0.59	0.91	0.21	0.87
Diffuse Lung Changes	0	0.81	0.56	0.96	0	0.99
Fibrosis	0	0.98	0.50	0.98	0	0.98
Chronic Lung Changes	0	0.99	0	0.99	0	0.96
Stasis/Edema	0.33	0.96	0.53	0.96	0.21	0.92
Costophrenic Angle Blunting	0.46	0.81	0.21	0.87	0	0.98
Pleural Effusion	0.47	0.91	0.86	0.94	0.74	0.87
Costophrenic Angle Blunting AND Pleural Effusion	0.64	0.81	0.71	0.82	0.72	0.86
Atelectasis	0.22	0.83	0.36	0.92	0.59	0.75
Pleural Changes	0	0.96	0.25	0.97	0.57	0.98
Former Operation in Lung Tissue	0	0.99	0	0.96	—	1
